# Implicit Statistical Learning Across Modalities and Its Relationship With Reading in Childhood

**DOI:** 10.3389/fpsyg.2019.01834

**Published:** 2019-08-22

**Authors:** Elpis V. Pavlidou, Louisa Bogaerts

**Affiliations:** ^1^Psychology in Education Research Centre, Department of Education, University of York, York, United Kingdom; ^2^Haskins Laboratories, Yale University, New Haven, CT, United States; ^3^Department of Psychology, The Hebrew University of Jerusalem, Jerusalem, Israel

**Keywords:** implicit statistical learning, artificial grammar learning, modality specificity, reading, reading fluency, children

## Abstract

Implicit statistical learning (ISL) describes our ability to tacitly pick up regularities from our environment therefore, shaping our behavior. A broad understanding of ISL incorporates a great range of possible computations, which render it highly relevant to reading. In the light of this hypothesized relationship, ISL performance was explored in young (M = 8.47 years) typical readers (*N* = 31) across three different modalities (i.e., visual, auditory, and tactile) using the Artificial Grammar Learning (AGL) paradigm. Adopting repeated measures and correlational designs, the obtained data revealed modality constraints: (1) above-chance performance was observed on the visual and tactile tasks but not on the auditory task, (2) there was no significant correlation of ISL performance across modalities, and (3) split-half reliability of visual and auditory tasks was reasonably high, yet for the tactile task it was close to zero. Evaluating the relation between ISL ability and language skills, we observed a positive correlation between visual ISL performance and phonological awareness. We discuss these findings in view of current perspectives on the nature of ISL and its potential involvement in mastering successful (i.e., accurate and fluent) reading.

## Introduction

He who thus considers things in their first growth and origin, [.] will obtain the clearest view of them.

Aristotle, ca.350 BC

It is catholically accepted that successful reading requires the development of a repertoire of skills that feed into both accuracy and fluency while failure to master such skills results in reading difficulties. Significant progress has been made in unpacking and understanding the key cognitive and linguistic factors that govern reading accuracy and more recently those involved in reading fluency (e.g., Pikulski and Chard, 2005). Successful reading is viewed here as a prototypical example of skill acquisition, which, similar to the acquisition and development of other skills, is supported by our ability to extract patterns and regularities from our environment (e.g., Kaufman et al., 2010).

In recent years, implicit statistical learning^1^ (ISL hereafter) that is our ability to pick up structure from our environment (over time) in an undirected fashion has emerged as a strong candidate mechanism to explain, amongst other, linguistic phenomena (e.g., Saffran and Wilson, 2003; Evans et al., 2009). This contemporary theoretical approach binds reading (and language acquisition overall) with a general (rather than language specific) capacity to detect, store, and use statistical regularities in the input (e.g., Arciuli and Simpson, 2012a, b; Frost et al., 2013; Erickson and Thiessen, 2015). As expected by statistical learning-based theories of language acquisition, ISL is established early in development (e.g., Saffran et al., 1996; Fisher and Aslin, 2002; Kirkham et al., 2002; Bulf et al., 2011): according to Goschke and Bolte (2007) the adaptation of our behavior to recurring and sequential patterns is a fundamental function of learning and thus, the encoding and exploitation of such regularities becomes an adaptive advantage (Conway and Pisoni, 2008).

Language constitutes a potent example of a learning environment that requires the exploitation of regularities: spoken words are characterized by idiosyncratic patterns of transitional probabilities (i.e., the conditional probability of one element given another element) that constrain their internal structure. Each writing system is then characterized by a set of correlations that determine the possible co-occurrences of letter sequences, and by high and low correlations of grapheme (letter) to phoneme (speech sound) mappings with different degrees of high and low correlations between letters and speech sounds characterizing different writing systems. Typically, regular letter to speech sound (L-SS) associations are taught to children explicitly (e.g., by giving examples and/or activities to reinforce them) (Apfelbaum et al., 2012). However, many L-SS correlations do not abide to simple rules and thus, are not explicitly taught; instead are picked up implicitly by the learner over increasing exposure to print (e.g., Cassar and Treiman, 1997; Pacton et al., 2001).

### ISL, Language Acquisition, and Reading

Starting from Saffran et al. (1996) seminal work on infants’ ISL abilities and amidst avid critics and unapologetic fans, the prominent role of ISL in spoken language acquisition has been firmly established over the past two decades (e.g., Saffran and Wilson, 2003; Evans et al., 2009). Studies on infant learning (e.g., Marcus et al., 1999; Clohessy et al., 2001) show that implicit learning abilities are already well established in infancy compared to other less well-developed explicit learning abilities at this age. ISL is viewed as the vehicle the novice learner is using to parse language (e.g., Saffran et al., 1996; Gomez and Gerken, 2000; Gómez, 2002; Kirkham et al., 2002); and it is most closely associated with tracking the sequential statistics (typically transitional probabilities) in the incoming speech stream.

However, a broad understanding of ISL and its’ link to language incorporates a great range of possible computations (e.g., frequency of individual elements, frequency of co-occurrence, distributional cues, etc., see Erickson and Thiessen, 2015, for a review), which render it highly relevant to reading as well. For example, similar to spoken language, written language contains different types and degrees of statistical information such as distributional cues and co-occurrence across domains for L-SS mappings and non-adjacent dependencies for grammar. Sperling et al. (2004) state that reading fluency in particular, is mastered with a mixture of explicit and implicit learning mechanisms even in languages that are highly regular in grapheme-phoneme correspondences. Connectionist models of language learning (e.g., Seidenberg and McClelland, 1989; Plaut et al., 1996; McClelland and Patterson, 2002; Harm and Seidenberg, 2004) together with neuroimaging data (see Sawi and Rueckl, 2019) bolster the argument that any attempt to attain successful reading involves ISL procedures. ISL could mediate abilities that are directly involved in reading such as phonological awareness (e.g., Spencer et al., 2015), accounting in turn for individual variation in children’s reading performance.

It is well documented that phonological awareness (part of phonological processing) is involved not only in L-SS mappings but also in reading comprehension by aiding phonological recovery during both reading aloud and silent reading (e.g., Ashby, 2006). ISL could shape not only visual word processing abilities but also the phonological representations and the automatic access of such representations in long-term memory via the “exploitation” of the regularities inherent in spoken and written language. Reasoning along these lines, Mainela-Arnold and Evans (2014) hypothesized that the ability to track statistical sequential regularities in speech streams may be critical to the acquisition of lexical-phonological knowledge and demonstrated a relationship between auditory ISL and lexical-phonological abilities in children with specific language impairment but also in children with typical development (ages 8–12): poor statistical learners, they found, were also poor at managing lexical-phonological competition. Relatedly, Spencer et al. (2015) tested, in a large sample of 4–10 year old children, ISL abilities and a series of tasks tapping constructs crucial to the development of early literacy skills: oral language skill, vocabulary knowledge and phonological processing. ISL abilities were measured with two different tasks: an auditory Saffran-style word segmentation task and a visual, interactive Simon-AGL task with colored squares after Conway et al. (2010). Using structural equation modeling, the authors revealed that generally speaking ISL accounted for a unique portion of the variance in these literacy-related skills. Interestingly, the two ISL tasks did not load onto a single latent variable and whereas words segmentation had a stronger influence on oral language skills, the visual Simon-AGL task had a stronger contribution to phonological processing skills. This result was interpreted in terms of the different SL mechanisms these two ISL tasks differentially tap into [in line with the extraction and integration framework put forward by Thiessen et al. (2013)]. Importantly, they also suggest that SL abilities in both the auditory and visual modalities are related to early literacy acquisition and that SL abilities in sensory modalities other than the auditory may play a role in the development of phonological skills (potentially because of shared underlying learning mechanisms).

A smaller set of studies on ISL and typical reading has demonstrated correlations between ISL abilities and reading skills in first (Arciuli and Simpson, 2012b; e.g., Apfelbaum et al., 2012; Qi et al., 2019; von Koss Torkildsen et al., 2019; but see Schmalz et al., 2019 for contrasting results) and second language (Frost et al., 2013). The majority of these individual differences studies indexed ISL by one non-linguistic visual segmentation task (Arciuli and Simpson, 2012b; Frost et al., 2013; von Koss Torkildsen et al., 2019), yet without the (explicit) assumption that the observed relationship is dependent on the visual presentation modality or the type of input statistics the task of choice taps on. In the theorizing, ISL is typically treated as a unified theoretical construct, a “*general capacity for picking up regularities*” that is predicted to correlate with measures of literacy (see Siegelman et al., 2017a, for a discussion).

### ISL: A Unified Construct or Not?

Originally, the domain-generality of ISL was invoked to argue against language modularity and innate theories of language acquisition (Chomsky, 1959; Fodor, 1984). The fact that ISL abilities were demonstrated in studies that used different types of stimuli including shapes (e.g., Pothos and Bailey, 2000; Pothos and Kirk, 2004; Bulf et al., 2011); alien figures (e.g., Arciuli and Simpson, 2012a); pure tones (e.g., Saffran et al., 1999); speech-like sounds (e.g., Gomez and Gerken, 2000) and syllables (e.g., Saffran et al., 2006); and tactile stimuli (i.e., finger vibrations) (e.g., Conway and Christiansen, 2005), let to the common belief that ISL is a unitary learning system (e.g., Bulf et al., 2011). Such unitary learning system is thought to execute similar computations across stimuli and sensory modalities (Frost et al., 2015).

The theoretically “appealing” view of ISL as a single entity is, however, challenged firstly by data from adult populations suggesting modality and stimulus-specific constraints (e.g., Conway and Christiansen, 2005, 2006; Mitchel and Weiss, 2011; see Frost et al., 2015, for a comprehensive review). A second finding that is puzzling for ISL as a unified construct is the virtually zero correlation between ISL performances in the auditory vs. visual modality (e.g., Siegelman and Frost, 2015). If there is something like a domain-general ISL faculty extracting patterns across modalities, why would someone who performs well on an ISL task with auditory stimuli not do well on an ISL task with visual stimuli also? These modality-specific effects were demonstrated predominantly in adult populations but a third piece of evidence comes from a cross-sectional study testing visual and auditory ISL performance of children at ages 5–12 (Raviv and Arnon, 2018). Whereas visual SL performance improved linearly with age, auditory SL performance, albeit lower on the average, was not superior for older children. What is the nature of ISL (in these young populations) that can explain differential developmental trajectories?

Recently, Frost et al. (2015) offered a theoretical framework reconciling domain-generality and specificity. ISL, they argue, is “*not a unitary mechanism, but a set of domain-general computational principles that operate in different modalities and, therefore, are subject to the specific constraints characteristic of their respective brain regions*” (p. 1). This framework raises an interesting question regarding the link between ISL ability and reading ability: Is the association underpinned by a shared reliance on the ability for registering the statistical properties of the input or rather driven by the ability of our visual system specifically to efficiently encode and effectively internally represent visual stimuli (see also Bogaerts et al., 2016)? In other words, are ISL abilities in modalities other than the visual also predictive of reading performance?

Qi et al. (2019) very recently explored the association between reading skills and both visual and auditory ISL with results suggesting that, maybe somewhat surprisingly, auditory ISL contributes more strongly to certain aspects of reading compared to visual ISL. Importantly, auditory ISL might be predictive of reading simply because it taps on the same domain-general capacity for picking up sequential regularities as the visual task or rather via its contribution to oral language skills (Spencer et al., 2015) and/or phonological processing abilities.

### The Present Study

In the light of sparse empirical data from young populations on the nature of ISL *per se* and the proposed mechanisms via which it could facilitate reading early in development, the purpose of this study becomes twofold: to explore on the one hand, whether ISL can be best described as a unified ability or as a constituent one [if one considers the different neurocognitive computations associated with how information is processed in specific modalities (Frost et al., 2015)] by looking at performance across modalities; and on the other hand, to systematically unpack the relationship of ISL with reading and reading-related abilities in childhood. Embracing, however, the possibility of ISL having both a general component and a domain-specific one, we aim to shed light on which component underlies the hypothesized relation with reading skill. To provide some answers to the aforementioned questions, the Artificial Grammar Learning (AGL) framework was used.

Artificial grammar learning (Reber, 1989) is a paradigm widely used for studying ISL and it has been used previously with young children (Pavlidou et al., 2009, 2010; Pavlidou and Williams, 2014). Its framework provides the theoretical and empirical grounds for exploring various hypotheses pertinent to how implicit learning mechanisms contribute to reading as it is thought to draw on the mechanisms that recognize complex statistical regularities (e.g., Petersson et al., 2004). In a typical AGL task participants are shown strings of letters that are constructed based on a particular rule system (artificial grammar) and then they are asked to identify from a novel set of strings those compatible with the old (Pothos and Bailey, 2000) that is to make grammaticality judgments. Various explanations are proposed to account for typical participants’ behavior during the AGL learning episode (see Redington and Chater, 1996; Pothos, 2007, for reviews). Participants are found to be sensitive to specific item factors such as the similarity of testing items to training items (Brooks, 1978; Brooks and Vokey, 1991; Vokey and Brooks, 1992), fragment (e.g., bigrams or trigrams) information (e.g., Perruchet and Pacteau, 1990, 1991; Witt and Vinter, 2011) and structure (rules or micro-rules) (e.g., Dulany et al., 1984; Manza and Reber, 1997). It is suggested that sensitivity to the level of associative strength of the test stimuli (chunk strength) to the training stimuli reflects a statistical fragment-dependent learning mechanism (chunking models of implicit learning, e.g., Boucher and Dienes, 2003 but see Pothos, 2007 for a comprehensive review of available AGL models). Sensitivity to structure (the system used to create both training and testing items) on the other hand is thought to indicate a structure-based acquisition mechanism (for rule-based models of implicit learning see e.g., Dulany et al., 1984; but see Pothos, 2007). These properties make AGL a suitable “analog” for some of the mechanisms that novice readers could capitalize on to master reading.

The choice of looking at ISL using the AGL paradigm across three different modalities and within subjects will add important behavioral data on whether this type of learning is served by a unified mechanism that “behaves” in a similar way across different *perceptual* and *item level* dimensions given that stimulus modality is thought to impact the learning process itself (Silva et al., 2018). Previous studies found a quantitative advantage of the auditory modality (e.g., Conway and Christiansen, 2005), however, recent data do not confirm modality differences in ISL but yet acknowledge the inherent constraints to each modality (e.g., Conway and Christiansen, 2009). AGL has the advantage of allowing experimental manipulations on the *type of information* (i.e., verbal/non-verbal), *modality* (i.e., visual, auditory, or tactile), and *item level* (e.g., adherence or not to the grammar rules or associative strength) all of which allow the formation of numerous testable hypotheses on the nature of the learning and its resulting knowledge. Every effort has been made to tightly control experimental procedures and materials across the senses: To ensure that we still have comparable input across senses but at the same time we have induced maximum learning by accounting for inherent modality constraints, the structure of the ISL stimuli is the same across sensory conditions but the presentation of ISL stimuli is spatial for the visual modality and temporal for the auditory (and tactile) modality. Following the work of Conway and Christiansen (2005, 2009) on typical adults, the present set of experiments provides an adequate comparison of learning across three modalities (vision, audition and touch) and an insight on how modality constraints might affect ISL early in development.

In turn, the combination of the AGL tasks with a range of reading and cognitive measures provides the platform to consider the relationship between ISL performance and reading in more detail. This is the first study to our knowledge that looked at ISL learning performance across modalities and its relationship with reading in young typical children. A robust test of the hypothesis that ISL is related to reading ability involves (1) selection of an ISL task with non-verbal stimuli that has no specific predefined relationship with reading processes *per se*, and (2) use of standardized tests of phonological awareness and reading ability that were not designed with the probabilistic link between letters and speech sounds, and among letters, in mind (Arciuli and Simpson, 2012b). This was the reasoning adopted in the present study.

## Materials and Methods

### Subjects

This study was carried out in accordance with the recommendations and approval of Yale University Human Investigation Committee (HIC) and the University of Edinburgh The Psychology Research Ethics Committee. Written and informed consent and assent were obtained from parents and the participating children, respectively.

Thirty-one^2^ typically developing children participated in the study and they received monetary compensation for their participation. They were between 6 and 9 years old (M = 8.47, SD = 1.19 years; F = 15, M = 16). Children did not have a reported history of reading, speech or hearing impairment.^3^ They all had normal or corrected to normal vision. All testing sessions took place at Haskins Laboratories facilities in New Haven, CT (see section “Procedure” for more details).

### Materials

#### Background Measures

Children received a battery of general and reading-related cognitive measures (see Table 1 for a summary of participants’ scores):

**TABLE 1:** Participants’ descriptive statistics on standardized behavioral measures, all reported in standardized scores.

**Measures**	**Range**	**Mean**	**SD**
WASI (II): Verbal comprehension	85–160	111.06	16.16
WASI (II): Perceptual reasoning	89–137	109.32	11.49
WASI (II): Full scale	93–139	111.26	11.14
TOWRE: Sight word	99–138	115.32	9.63
TOWRE: Phonemic decoding	97–137	115.39	12.60
TOWRE: Composite score	98–137	118.36	12.65
WJ-III: Broad reading	98–135	115.96	10.27
WJ-III: Basic reading	97–136	118.30	10.38
WJ-III: Reading fluency	90–146	113.07	14.07
WJ-III: Spelling	96–136	117.28	13.03
CTOPP: Phonological awareness	84–137	110.10	16.18
CTOPP: Phonological memory	76–144	111.24	17.77
CTOPP: RAN	88–122	103.50	11.98

##### General intellectual ability and memory

General intellectual ability was assessed using the Wechsler Abbreviated Scale of Intelligence (WASI II) (Wechsler, 2011) for verbal comprehension and perceptual reasoning. Children’s working memory was assessed using the Digit Span subtest from CTOPP 2 (Wagner et al., 2013).

##### Literacy

###### Reading and spelling

To evaluate reading skills, the 3rd edition of the Woodcock–Johnson Test of Achievement (WJ-III; Woodcock et al., 2001) was administered. We measured the Broad Reading Composite (WJBR) score, which was a composite of scores on the following subtests: Letter-Word Identification (recognizing letters and reading real words of increasing difficulty), Reading Fluency (speeded reading of sentences), and Passage Comprehension (reading and understanding short passages). We also calculated the Basic Reading and Reading Fluency composite scores. Children also completed the Test of Word Reading Efficiency (TOWRE; Torgesen et al., 1999) (speeded reading of single words and non-words), which is composed of two subtests: the Sight Word Efficiency (SWE) and Phonetic Decoding Efficiency (PDE). We computed both sub-scores as well as the composite score. Finally, children’s spelling ability was measured using the Spelling subtest from WJ-III.

###### Phonological processing and rapid automatized naming (RAN)

To tap phonological awareness, we used the Elision and Blending Words subtests from the Comprehensive Test of Phonological Processing 2 (CTOPP 2) (Wagner et al., 2013). We also used from the same test battery, the composite score for Memory for Digits and Non-word Repetition subtests to measure phonological memory and RAN for digits to measure RAN.

#### The Implicit Statistical Learning Tasks

##### Apparatus

Three AGL tasks were developed in which information and item levels were kept constant but introduced via a different modality (visual, auditory and tactile). In all three modalities the stimuli were non-verbal: (1) The visual task used unfamiliar shapes; (2) The auditory task used pure tones; (3) The tactile task used finger vibrations. Based on adult data (Conway and Christiansen, 2005) suggesting that participants learn the predictive dependencies better when the visual stimuli are presented in simultaneous (spatial) fashion as opposed to sequential, it was decided to present the visual stimuli spatially (as opposed to auditory and tactile stimuli, which were introduced sequentially/temporally) to induce maximum learning.

Each task consisted of a training phase, which exposed the children to stimuli that followed the permissible transitions of the grammar (i.e., they were not random) and a test phase. Presentation order for both training and testing stimuli was randomized for each participant. The testing stimuli controlling for (a) adherence to the grammar rule and (b) fragment familiarity (*item level*) shared with the training items. By manipulating both grammaticality and chunk strength we can learn about the learning mechanisms children employ (in each of the modalities).

###### Visual non-verbal task (VNT)

**FIGURE 1:**
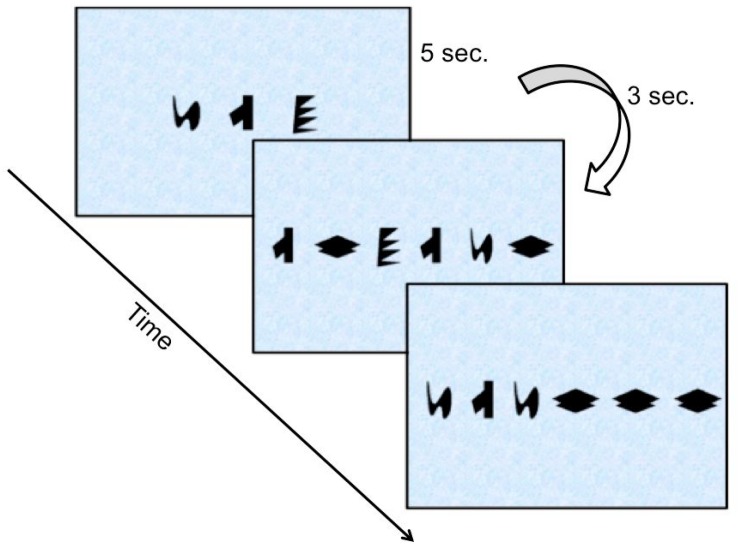
Examples of grammatical sequences children were shown during the training phase [individual shapes adapted with permission by Fisher and Aslin (2002) and Conway et al. (2007)].

The visual task was based on Knowlton and Squire (1996) experimental grammar. Children were trained on 69 grammatical sequences (i.e., sequences that followed the rules of the grammar) composed of two to six items, i.e., “alien” shapes (see Figure 1, for some examples of sequences); but they were not informed about the structured nature of the sequences. The sequences (irrespective of their length) were presented one at a time and remained on screen for 5 s (inter-sequence interval: 3 s). Children were advised to give their utmost attention and tap^4^ their hand whenever they noticed a new sequence on the computer screen.

After the end of the training phase, children were informed that the sequences followed some very complicated rules and that they had to choose from a new set of sequences those that seemed to follow the same rules or looked “familiar” to them with a verbal response (yes, if a sequence looks familiar to them; no, if it doesn’t look familiar). They were shown 32 novel test sequences (ranging between 2 and 6 items long). Presentation parameters here were identical to those in the training phase. Half of the sequences obeyed the rules of the grammar and were thus, labeled grammatical (GR) while the remaining half violated those rules and were labeled ungrammatical (UG). We also manipulated *associative strength* (referred to as “chunk strength” hereafter) to the training sequences, so that half of the GR items had high chunk strength (HCS) and half of the UG items had low chunk strength (LCS). Chunk strength for each sequence was calculated by dividing the total number of bigrams and trigrams (i.e., chunks) it consisted of with the total number of times the same chunks had appeared during the training. Note that children’s responses are scored according to grammaticality only: accepting GR items and rejecting UG items are correct responses, rejecting GR items and accepting UG items are incorrect responses.

###### Auditory non-verbal task (ANT)

The auditory task was designed by substituting the “alien” shapes with “alien” sounds that is pure tones (1 = 261.6 Hz, 2 = 277.2 Hz, 3 = 349.2 Hz, 4 = 370 Hz, and 5 = 493.9 Hz) using E-Prime^5^; the same training and testing items used for VNT were used to create the auditory stimulus set: the duration of each tone was 500 ms and it was introduced every 100 ms. After the end of one sequence of tones, there was an interval of 1700 ms followed by a fixation cross on the computer screen, which marked the beginning of a new sequence. Again, children were not informed about the structured nature of the tone sequences but were advised to pay attention to the “alien” sounds. They were then introduced to new tone sequences and were asked to indicate/decide which items sounded similar and which did not by using button presses.

###### Tactile task (TT)

The tactile task was designed as follows: a number was assigned to each of the four letters of the grammar and subsequently, each number was mapped on a specific finger [Knowlton and Squire, 1996 grammar had four positions so the last finger (pinky) was mapped onto 0 and did not correspond to any vibration]. Following this “coding scheme,” the letter items comprising the training and testing set used for the VNT were replaced with their corresponding fingers (see **Box 1**).

**BOX 1:**
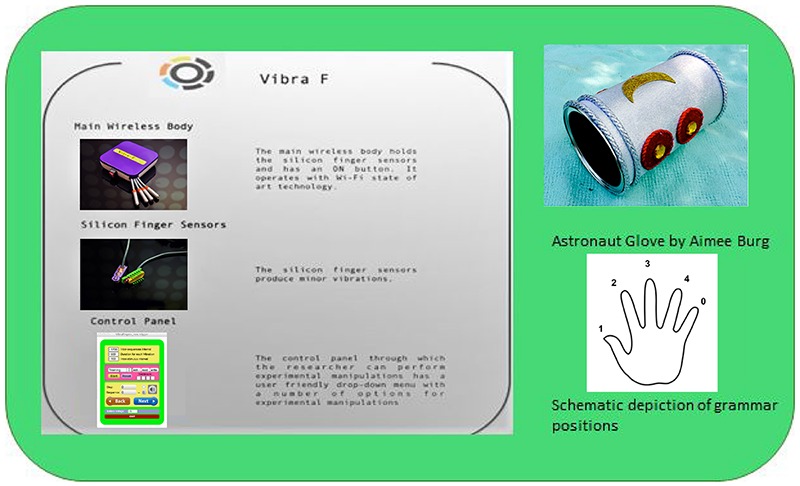
Tactile experiment materials. ©Elpis Pavlidou.

Using an innovative tactile device (see **Box 1**), minor finger vibrations were produced to each finger (i.e., fingers 1, 2, 3, and 4) during training and testing. The duration of each vibration was 500 ms and it was introduced every 100 ms. After the end of one sequence of vibrations, there was an interval of 1700 ms before a new sequence was introduced. Again, the same design as in the VNT and ANT tasks, respectively was used for the tactile task (TT). To impede interference from the other senses, children were asked to wear an “astronaut glove” (see **Box 1**) and headphones playing white noise for the entire duration of the experiment.

### Procedure

Children performed individually the tasks during three experimental sessions (within a span of ∼1–4 weeks apart; M interval = 2.2 weeks) with the following order of administration were possible: Session 1, WASI II/Visual Non-verbal Task (VNT)/TOWRE; Session 2, Hearing Test/Auditory Non-verbal Task (ANT)/WJ-III; and Session 3, Tactile Task (TT)/CTOPP 2.

## Results

To recap, the current tightly controlled experimental design explored (1) whether AGL learning in one modality is linked with learning in other modalities thus, pointing to the existence of a domain-general ISL mechanism; (2) what are the different statistics (induced/encouraged/tested by our experimental tasks across the different modalities) that helped children to learn the inherent regularities of our stimulus set; and (3) what is the relationship, if any, of ISL in the different modalities with our chosen standardized measures of reading and reading-related abilities.

### AGL Learning

**FIGURE 2:**
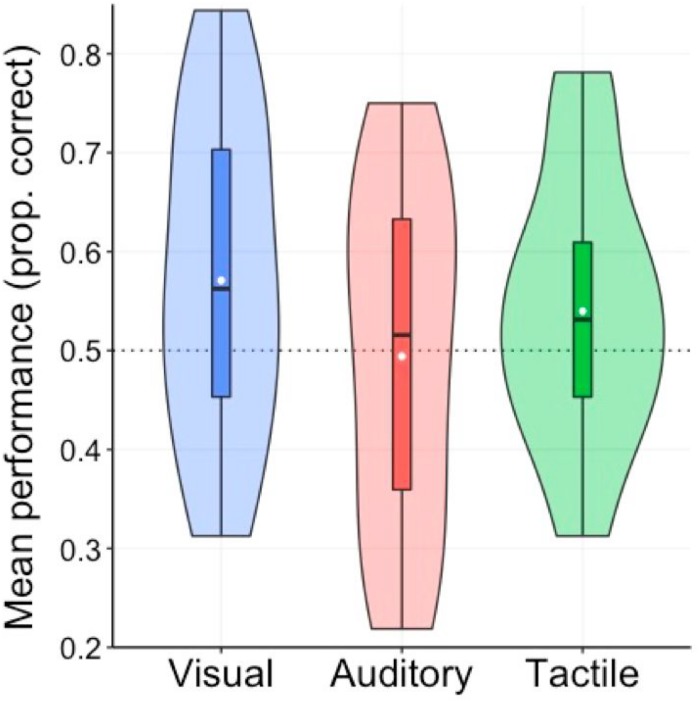
Performance on the ISL tasks in the three modalities. White disks show the means and midlines represent medians. Box limits indicate the 25th and 75th percentiles and whiskers extend to minimum and maximum value. The shape around each boxplot reflects the kernel probability density at the different levels of performance.

Figure 2 depicts children’s mean correctness on the three AGL tasks (R Core Team, 2019). An independent *t*-test was used to compare performance against chance level (0.5 or 50%) in all three conditions/experiments given that above chance performance in AGL literature is taken as an indicator of learning taking place. Children performed at above chance in the visual and tactile tasks but not in the auditory one (visual: M = 56.45%, SD = 0.13, *t*(30) = 2.72, *p*_adj_ = 0.01, *d* = 0.53; tactile: M = 55.34%, SD = 0.10, *t*(30) = 2.94, *p*_adj_ = < 0.01, *d* = 0.50; auditory: M = 49.48%, SD = 0.16, *t*(29) = 0.22, *p*_adj_ = 0.57, *d* = −0.03, with *p*-values adjusted using Holm’s correction for multiple comparisons). We further analyzed the data with a repeated-measures ANOVA with mean correctness as the dependent variable and *Modality* (visual vs. auditory vs. tactile), *Grammaticality* status (grammatical vs. ungrammatical) and the two-way interaction *Grammaticality:Modality.* A marginal main effect for Modality (*F*(2,58) = 2.50, *p* = 0.09, η^2^*p* = 0.08) suggests again modality differences, and we also found a significant main effect for Grammaticality (*F*(1,29) = 7.37, *p* = 0.01, η^2^*p* = 0.20), indicating that, across modalities, children provided more correct responses by correctly rejecting UG items (M = 57.47%, SD = 0.10) than by correctly accepting GR items (M = 50.14%, SD = 0.13). The interaction effect was not significant (*F* < 1, *p* = 0.53, η^2^*p* = 0.02).

### What Was Learnt During AGL?

The balanced design applied to the AGL test materials (in terms of the grammaticality and chunk strength of the items), allowed the exploration of structural (i.e., grammaticality status) vs. familiarity-based (i.e., chunk status) effects in the visual and tactile modalities (where we observed above-chance performance). We ran a Repeated-measures ANOVA with mean acceptance rate as the dependent variable and as predictors *Modality* (visual vs. tactile), *Grammaticality* status (grammatical vs. ungrammatical), *Chunk strength* (high vs. low), the two-way interactions *Grammaticality:Modality, Chunk strength:Modality, Grammaticality:Chunk strength* and finally the three-way interaction *Grammaticality:Chunk strength:Modality.* Note that if children’s acceptance responses are driven by grammar-structure/rule leaning we expect an effect of grammaticality, whereas a reliance on item familiarity irrespective of grammaticality status would produce an effect of chunk strength. If both learning mechanisms play a role then we should see an interaction effect between grammaticality and chunk strength.

**FIGURE 3:**
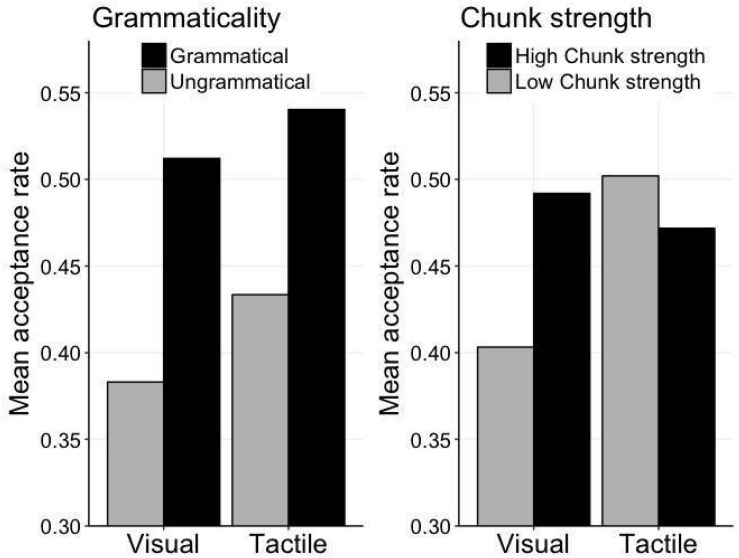
Illustration of the effects of grammaticality and chunk strength on acceptance rate for the ISL tasks in the visual and tactile modalities.

Our results revealed a main effect of *Grammaticality* (*F*(1,30) = 18.83, *p* < 0.001, η^2^*p* = 0.39) indicating, in line with the results above regarding mean correctness, higher acceptance rate for GR items relative to UG items. More importantly, we also observed a significant interaction between *Chunk strength* and *Modality* (*F*(1,30) = 4.52, *p* = 0.04, η^2^*p* = 0.13) and a three-way interaction between *Grammaticality, Chunk strength*, and *Modality* (*F*(1,30) = 5.88, *p* = 0.02, η^2^*p* = 0.16). This three-way interaction is illustrated in Figure 3. What we can infer from the figure is that whereas for the visual modality both grammaticality and chunk strength lead to a higher acceptance rate, for the tactile modality only grammaticality has such positive effect on acceptance rate (i.e., correctly accepting GR sequences and correctly rejecting UG sequences).

### Does AGL Performance Correlate Across Modalities?

**FIGURE 4:**
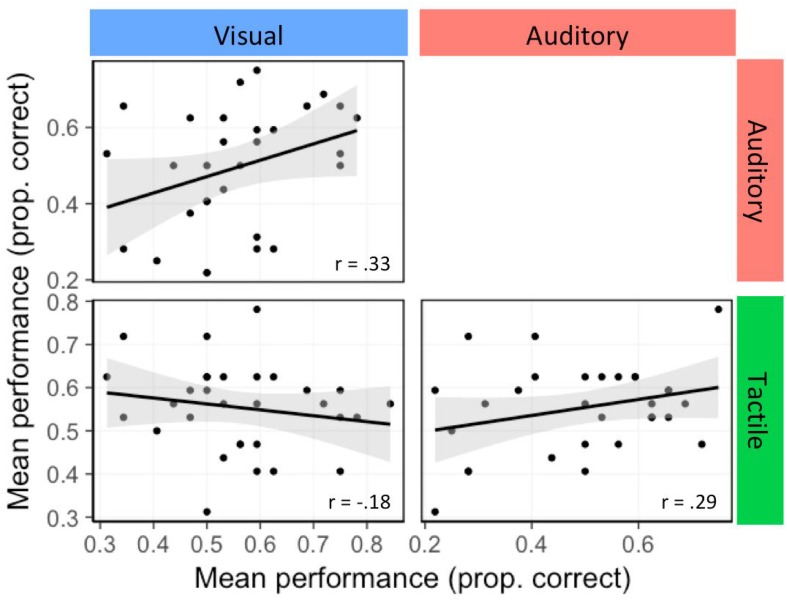
Correlations between task performance on the ISL tasks in the three modalities. Black lines represent regression lines and gray bands around them represent the standard error.

To determine the relationship, if any, of ISL performance across the three modalities, correlation analysis was applied. A Pearson correlation coefficient was computed to assess the relationship between performances across modalities (see Figure 4). There were no significant correlations (visual-auditory: *r* = 0.33 with CI95 = [−0.11 0.67], *p*_adj_ = 0.21; visual-tactile: *r* = −0.18 with CI95 = [−0.50 0.19], *p*_adj_ = 0.34; auditory-tactile: *r* = 0.29 with CI95 = [−0.14 0.62], *p*_adj_ = 0.25, *p*-values and confidence intervals adjusted using Holm’s correction for multiple comparisons).

Confidence intervals for all estimated correlations include 0 but are wide, hence we report also Bayes factors (BFs)^6^, which can help determine whether these non-significant results support the null hypothesis, or whether the data are rather just insensitive (Dienes, 2011). The strength of evidence for one hypothesis (here, the null hypothesis that the correlation is zero) compared to a competing hypothesis (here, the alternative hypothesis that the correlation is positive) is by convention considered moderate if the BF is larger than 3 (e.g., Jeffreys, 1961; Lee and Wagenmakers, 2013). For the correlation between visual and tactile performance we observe such moderate evidence with a BF0+ = 8.20, indicating that the data are about eight times more likely to have occurred under the null hypothesis than under the alternative hypothesis. The other two BFs are, however, smaller than 1: BF0+ = 0.48 for rvisual-auditory and BF0+ = 0.77 for rauditory-tactile, indicating inconclusive to weak evidence for a positive correlation.

Since the correlation between two measures is upper-bounded by their reliability, we also evaluated the split-half reliability of each of the tasks. Split-half reliability was obtained by correlating performance on odd and even test trials. Reliability correlations were found to be reasonably high for the visual (*r* = 0.42 with CI95 = [0.21 0.63], Spearman-Brown corrected = 0.58) and auditory (*r* = 0.56 with CI95 = [0.38 0.73], Spearman-Brown corrected = 0.72) tasks, but substantially lower for the tactile task (*r* = 0.16 with CI95 = [−0.08 0.41], Spearman-Brown corrected = 0.25). These reliability estimates assure us that the lack of correlations between the three tasks is not just the result of a lack of reliability but rather points to modality specificity, at least to some extent. Moreover, the numerically lower split-half in the tactile task suggests that the psychometric properties of AGL tasks with the exact same underlying grammar are not identical and possibly point again to an important constraint of the sensory modality an artificial grammar is learned and/or tested in. This result should be interpreted with caution though since the confidence intervals for the tactile split-half correlation and those for the other modalities do overlap.

### Does AGL Performance Correlate With Reading Measures?

Evaluating the link between ISL performance as measured in our three tasks and reading, we were interested in two theoretical connections^7^, the connection with (1) *phonological awareness*, (2) *basic reading skills*, and (3) *reading fluency.* Note that for phonological awareness and basic reading we simply used the standard score of the CTOPP 2 and WJ-III basic reading, respectively. For fluency, we averaged the TOWRE total standard score and the WJ-III fluency subtest as these both tap speeded reading. The use of standard scores (with ages norms) is particularly important because our participants ranged between 6-9 years of age, a dynamic age for language and early reading development.^8^

**FIGURE 5:**
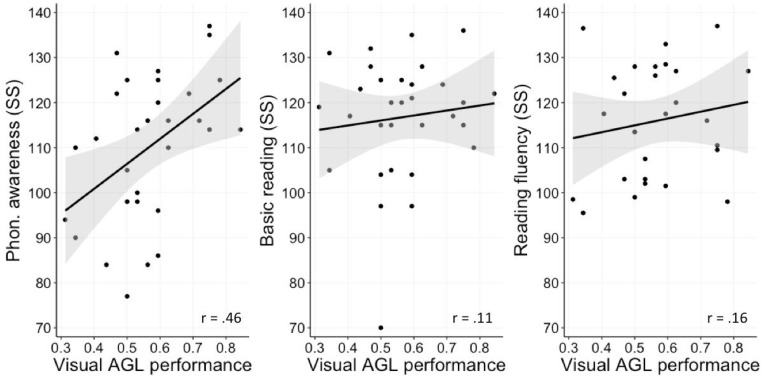
Correlations between task performance on the visual task and standard scores on three reading-related measures: phonological awareness, basic reading, and fluent reading (all in standard scores). Black lines represent regression lines and gray bands around them represent the standard error.

Based on this lack of significant correlations across the three AGL tasks and the low split-half reliability of the tactile task^9^ we focused on the visual and auditory AGL task and looked at them separately. All observed correlations between performance on the visual task and our reading-related measures were positive yet relatively small (see Figure 5) and only the correlation between phonological awareness and visual AGL performance was significant applying Holm’s correction for multiple comparisons (*r* = 0.45 with CI95 = [0.04 0.74], *p*_adj_ = 0.03). Controlling for general intelligence we found a partial correlation coefficient of *r* = 0.52 (*p* < 0.01). The non-significant Pearson correlation between visual AGL performance and basic reading was estimated *r* = 0.11, with CI95 = [−0.25 0.45] (*p*_adj_ = 0.81) and similarly, for AGL performance and reading fluency *r* = 0.16, with CI95 = [−0.28 0.55] (*p*_adj_ = 0.81).

**FIGURE 6:**
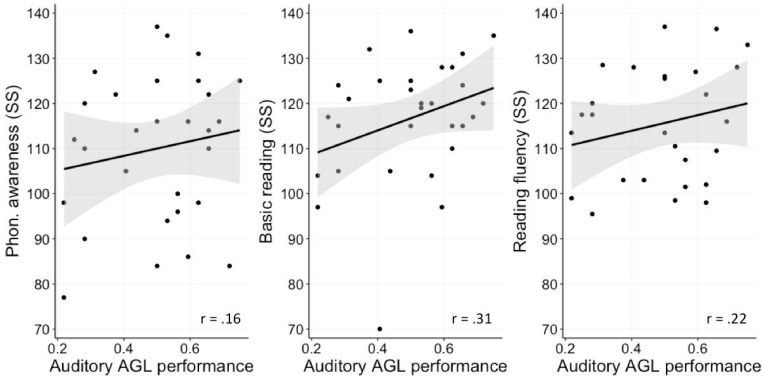
Correlations between task performance on the Auditory task and standard scores on three reading-related measures: phonological awareness, basic reading, and fluent reading (all in standard scores). Black lines represent regression lines and gray bands around them represent the standard error.

Finally, all correlations between performance on the auditory task and our reading-related measures were positive yet relatively small (see Figure 6) and none of those was significant (visual AGL-Phon. awareness: *r* = 0.16 with CI95 = [−0.22 0.50], *p*_adj_ = 0.53; visual AGL-Basic reading: *r* = 0.31 with CI95 = [−0.14 0.66], *p*_adj_ = 0.27; visual AGL-Reading fluency: *r* = 0.22 with CI95 = [−0.23 0.59], *p*_adj_ = 0.53).

Given that – with the exception of the correlation between visual AGL performance and phonological awareness – correlations were non-significant, we report again BFs. Table 2 shows that all BFs for the correlations reported above as non-significant fall within the range between 0.33 and 3 and are hence considered as inconclusive, or only weak evidence for either hypothesis.

**TABLE 2:** Bayes factors (BF0+) for each of the correlation pairs, quantifying the strength of evidence for the null hypothesis that the correlation is zero compared to the alternative hypothesis that the correlation is positive.

	**Phonological awareness**	**Basic reading**	**Reading fluency**
Visual AGL	0.10	2.66	1.94
Auditory AGL	2.01	0.60	1.35

## Discussion

Based on the hypothesized link between ISL and proficiency with written language, we explored in this study whether the ability to detect structure and fragment correlations implicitly in non-verbal spatial (i.e., visual symbols) or temporal (i.e., pure tones) arrays would correlate with phonological awareness and performance in reading measures of accuracy and fluency. In parallel, we were interested in investigating the nature of the ISL (whether domain – general or specific) given that it is still hotly debated in the literature. Hence, we looked at children’s performance across three modalities, i.e., vision, audition and touch. We also investigated which type of knowledge children acquire and base their familiarity judgments in the test phase of an AGL task (i.e., knowledge of the underlying grammar (rules) or chuck strength).

To summarize our results, we found:

(1) Above-chance performance on the visual and tactile AGL tasks but not on the auditory task with pure tones.
(2) No significant correlations between performances on the AGL task across the different modalities. Combined with the observation of reasonably high split-half reliability for the visual and auditory tasks this suggests at least some degree of modality specificity.
(3) Positive albeit small correlations between visual AGL performance and phonological awareness, basic reading and fluent reading. Only the correlation with phonological awareness reached significance. Similarly positive small correlations were observed between auditory AGL performance and our three reading-related measures but none of those were significant.

### ISL Performance Across Domains

First, we explored whether children are able to show learning when faced with unfamiliar training items during short exposure times and novel testing items. Children were able to show above-chance ISL for visual, spatially arrayed, input but also for tactile, temporally presented, input confirming findings from adult populations (Conway and Christiansen, 2005). No other studies to our knowledge have demonstrated ISL abilities in touch in young populations, namely children. Contrary to adult data and the perceived supremacy of the auditory modality in ISL, children did not show learning for auditory input. In fact, there was a numeric advantage of the visual modality. Although authors suggest that human visual statistical learning is similar to auditory learning (e.g., Aslin et al., 1998; Fiser and Aslin, 2002; Kirkham et al., 2002), such conclusions were based on studies that did not use comparable stimuli or procedures across modalities. Considering that our training and testing materials were identical in terms of their underlying structure (i.e., we the same training and testing items across modalities that we developed based on the same grammar) we can make more “refined” comparisons across modalities. Following this, we acknowledge that there are similarities in how infants and adults learn across modalities but the lack of evidence for learning in the auditory modality in our data provides a first piece of evidence suggesting potential important differences in children’s learning that should be further explored using other ISL paradigms.

### One Modality Is Not the Other

The lack of correlation of performance across modalities (with one out of the three BFs proving moderate evidence in favor of the null hypothesis) further enhances our argument and advocates for potential important learning differences across modalities: ISL does not appear as a unified entity but rather as subject to modality constraints in childhood, confirming data from adult populations (Siegelman and Frost, 2015): a child’s performance in one modality might not generalize to other modalities, rather children may be good in detecting structure and/or fragment correlations in one modality (with one type of stimuli) but not in another (with another type of stimuli). The lack of correlation of performance across modalities was observed despite a reasonable split-half correlation for the visual and auditory tasks. The tactile task by contrast displayed a very low split-half reliability, even with above-chance performance. The differential psychometric qualities of the same test in different modalities is interesting by itself and potentially further attests to modality differences and constraints. Note that split-half reliability is an important type of reliability but concerns only the internal consistency of the measure and not its stability in time. For a full evaluation of reliability one would need to also investigate test-retest reliability, which is typically lower.

Overall, our data supports the finding that ISL as measured by AGL taps on mechanisms that discover both structure and fragment information (e.g., Knowlton and Squire, 1996; Pavlidou et al., 2010; Pavlidou and Williams, 2014). Yet again, the sensitivity children showed toward both the grammatical structure and chunk strength for the visual stimuli and the sensitivity to grammaticality only for the tactile stimuli provides evidence for such domain-specific constraints on learning mechanisms.

### Is ISL Associated With Phonological Awareness and Reading?

Importantly and pertinent to our main theoretical question, we explored the relationship of ISL (as tested by AGL) with phonological awareness, basic reading as well as fluent reading in typically developing children.

The first striking finding is that children who performed well in the visual task, that is, appear to have picked up the implicit structure embedded in the spatially presented visual shapes, on average, scored well on the phonological awareness task (as tested by CTOPP 2). Good phonological awareness is pivotal to the development of accurate and fluent reading as it encapsulates the novice reader’s ability to map letters onto their corresponding speech sounds. However, given the arbitrary L-SS mappings in English (and other deep orthographies) where one letter has more than one speech sound mappings, efficient associations are thought to be the result of both explicit and implicit learning processes. Our findings bolster this argument by adding important data on the potential link of ISL with efficient reading in childhood by presenting a positive trend between visual ISL and phonological awareness. ISL could be a key mediating factor, a mechanism that facilitates the novice reader in picking up not only the regular but importantly the irregular L-SS mapping, resulting to fast and effortless word retrieval. What is surprising, however, is that we did not observe a (significant) correlation between performance on the auditory task and phonological awareness. As we discuss in the section below one possibility is that such a correlation does exist but given measurement error and our relatively small sample our study could not reveal it. This remains, however, an open question for future research.

Although we observed small positive correlations between both basic and fluent reading and AGL performance those were not found to be significant. BFs for the non-significant correlation pairs all fell within the range between 0.33 and 3, leading to the conclusion that the data – rather than providing substantial evidence for the null hypothesis – are uninformative about whether the null or the hypothesis of a positive correlation was supported. Whereas the theoretical link detailed in the introduction would definitely have predicted a positive relationship not only between AGL performance and phonological awareness but also between AGL performance and reading skills, our result is in line with previous studies linking individual differences in ISL performance with individual differences in linguistic skills in children, typically reporting correlations which do not exceed *r* = 0.30 (e.g., Arciuli and Simpson, 2012b; Shafto et al., 2012; Mainela-Arnold and Evans, 2014; Spencer et al., 2015). It is worth noting that our split-half reliability was far from perfect, which is typical not just for AGL but for many different tasks indexing ISL (e.g., Siegelman et al., 2017a; Bogaerts et al., 2018; West et al., 2018). Since the correlation between two measures is upper-bounded by their reliability, these weak correlations could in fact reflect a stronger true correlation (Bogaerts et al., 2018; Conway et al., 2019).

Our result on phonological awareness is in line with Spencer et al. (2015) findings on early reading skills and statistical learning that confirmed the relation between ISL and phonological processing (which includes phonological awareness) using a large sample. Taken together, our findings and Spencer et al. (2015) findings suggest that ISL supports reading-related skills such as phonological awareness both at the early and later stages of mastering. Moreover, the correlation between visual AGL performance and phonological awareness remained significant also when controlling for age or general intelligence, which suggests that there is a “legitimate” link between visual ISL (as manifested in AGL and reading-related skills). Data from reading abilities in adult self-paced reading (Misyak and Christiansen, 2011) and adult second language learning (e.g., Frost et al., 2013) do suggest that this link persists also for later reading, although this clearly requires further investigation.

### Limitations and Directions for Future Research

Our study is based on a relatively small sample size (*N* = 31) and this fact, admittedly, raises the concern of low power, and potentially missing correlations that are in reality present (and less accurate estimations of the correlation sizes in general). That the correlations with reading in the current study were positive but not significant, with BFs indicating that the data are insensitive rather than supporting the hypothesis of a zero correlation, calls for future work with larger developmental samples. A power analysis (G^∗^Power 3.1, Faul et al., 2009) assuming an expected effect size of 0.30, a desired power of 0.90 and a one-tailed test, recommends a sample size as large as 88. Such large sample sizes are definitely a challenge in developmental research (and even more so in multi-session experiments) but they prove to be highly necessary. Future studies could also focus on a more restricted age group; whereas in the current investigation we employed standard scores (with age norms) to control for age we cannot exclude the existence of developmental effects and our test group is not sufficiently large to systematically explore them. Note that with a limited sample, results may also be more affected by deviant observations. The scatter plot depicted in Figure 5 demonstrates, however, that this does not seem to be the case.

Another point to consider is that even in our visual and tactile tasks (for which we observed an above-chance mean group performance) respectively 39 and 29% of children did not perform above chance and hence did not display evidence for learning. This pattern of results is very common for both developing and adult samples (e.g., Don et al., 2003; Gabay et al., 2015, see also Siegelman et al., 2017a). From an individual differences viewpoint at-chance performance might be meaningful, yet a substantial proportion of the data points simply reflect noise in terms of predictive validity (see Siegelman et al., 2017a; see also Siegelman et al., 2017b for a discussion on issues arising from looking at individual differences in statistical learning). Future studies might hence want to develop methods optimized for the measurement of individual differences in developmental samples. Another possible approach would be to explore correlations looking only at the subset of children who show evidence for learning but this would require a larger sample than the one we had available in the present study.

A third point that deserves some attention is the distinction between modality and the specific stimuli we choose to employ. The use of non-verbal stimuli (e.g., pure tones) in our experiments have the advantage of inducing the net efficiency of ISL computations, however, as we used one type of stimulus in each modality we cannot distinguish modality effects from stimulus effects with the use of a specific type of stimulus. It would be therefore interesting to explore in future work stimulus and modality effects by using multiple types of stimuli within the same perceptual dimension (e.g., for the auditory domain pure tones and non-verbal daily sounds, Siegelman et al., 2018).

Finally, the low reliability of the tactile AGL task calls our attention to the mandatory pursuit of exploring the extent to which the various available paradigms are robust proxies of ISL. Therefore, subsequent studies should enhance our understanding of the psychometric properties of all available ISL tasks to inform theory and guide research practice.

## Conclusion

On the whole and from a theoretical point of view, our data on AGL performance across perceptual modalities suggests notable modality differences and constraints in the implicit assimilation of statistical regularities. For the types of stimuli and the underlying grammar this investigation tested, we found that young children (6–9 years old) perform, as a group, above-chance performance on a visual task with abstract shapes and tactile tasks with finger vibrations, but not on an auditory task with pure tones. Moreover, we observed no significant correlation of ISL performance across modalities and suggestive differences in the psychometric properties of the different tasks.

Despite such modality difference there might be shared computational principles for the extraction of statistical information (adjacent/non-adjacent dependencies) that operate in different modalities (Frost et al., 2015) and these could be implicated also in reading-related skills (Frost et al., 2013), given that statistical regularities are inherent to each language system. Our finding of a significant positive correlation between visual AGL performance and phonological skills provides support for such a theoretical link. However, at the same time we observed surprisingly low and non-significant correlations between AGL performance and our measures of basic reading as well as reading fluency. These could indicate developmental effects; yet they could also be the result of measurement limitations or the combination of both. Nevertheless, neuroimaging data (e.g., Kosslyn and Koenig, 1992; Paquier and Mariën, 2005; Shohamy and Turk-Browne, 2013) suggest that successful reading is driven by an interaction between domain general and domain specific mechanisms, which support not only efficient learning of perceptual features but also implicit statistical regularities idiosyncratic to each language. Clearly however, we are in need of additional larger-scale systematic investigations of ISL skills and reading skills at both the behavioral and neurobiological levels of analysis and across various populations.

## Ethics Statement

This study was carried out in accordance with the recommendations of Human Investigation Committee (HIC), Yale University, and the Psychology Research Ethics Committee, University of Edinburgh, with written informed consent from all subjects. All subjects gave written informed consent in accordance with the Declaration of Helsinki. The protocol was approved by HIC (Protocol No. 1304011782).

Research conducted with children at Haskins Laboratories, Yale University, and Edinburgh University was subject to and covered by human subjects protocols and institutional review boards, respectively. The policies that are in place define the standards for the participation of children in research studies conducted at or by Yale University, The University of Edinburgh, and partner institutions. As stated in Yale Institutional Review Boards protocol “children participating in research constitute a special class of subjects for which special protections apply… All children considered for enrollment in, or enrolled as subjects in research must be treated in a manner commensurate with their special status as minors. Such research must be designed to ensure the appropriate enrollment of children and employ additional safeguards as described in this policy to ensure and protect their rights and welfare.”

The research team made sure that all children’s rights were met and that all the experimental conditions were age appropriate so that children benefit the most from the inherent procedures and the overall experience of participating in psychological research. It was of profound importance and an urgent priority of the research team to guarantee maximum research quality as this is defined and understood in ethical codes of research practice. Thus, following the ethical research guidelines for children as participants, all ethical issues were addressed successfully. More specifically, a number of essential criteria for good practice were adopted in relation to the study:

### Process of Consent/Assent

Initially, a letter was drafted and sent out to parents/guardians/caregivers seeking written permission for the child to participate in the research. The parental permission form was written in a language understandable by the parent/guardian/caregiver and contained all elements of informed consent, including a description of the research study, the research procedures and any potential risks or benefits (a copy of the form can be provided upon request). The default mode of informed consent was “opt-in”: all consent forms contained two parts: an explanatory statement and the consent form (which was signed). The signed part asked the parent/guardian/caregiver to agree on their child’s participation by signing the form and returning the signed part to the teacher and/or researcher. This way the parent/guardian/caregiver was actively giving consent for participation.

Further to parental consent, we asked for child consent either through writing or oral consent in case of poor literacy skills. We also created information sheets to provide more detailed information on the experimental conditions and procedures relating to the study. Given that the study was targeting children of different age groups, we developed separate age-appropriate information sheets to ensure that all children fully understand what they were giving consent for. Additionally, prior to giving their consent, parents and children were thoroughly explained what the tasks entail and what they are expected to do during those tasks. This ensured that both children and parents/caregivers have a good understanding of the experimental procedure and their input during this process and that they are fully aware about the content of their consent. Despite parental/guardian and child consents, children were frequently reminded that they can opt out of any experiment and at any given time point during the project without any adverse effects or modifications in compensation. In more detail:

#### Evaluation of Subjects Capacity to Provide Informed Consent/Assent

For children, parent/guardian/caregiver (s) were asked to sign the consent forms to allow their children to participate in the study. Parents were always encouraged to ask questions about each study or the consent form itself before they sign. As a matter of course and as stated earlier, children who could follow verbal instructions were asked to provide written (were possible) or oral assent before they participate. Children were given a brief introduction to the tasks so that they ascertain explicitly that they are happy to take part. Children were told that they do not have to complete the tasks if they do not wish to and that they can choose to stop doing them at any time. Given that participation in research is voluntary, children had the right to withdraw at any time. Because of the possibility that children may not be able to communicate their desire to withdraw so clear, the research team took up the responsibility to listen to them and be prepared to have to stop a session prematurely. Children were taken seriously when they began to show signs of discomfort or say “no.” Nevertheless, the research team made every effort to make the child feel comfortable during the consent process (procedures discussed above) as well as during the study. All children were asked to summarize what they have been told about what they will be doing during each experiment to ensure that they comprehended the procedures.

#### Safety and Data Monitoring Plan

The assessment of the overall risk level for children participating in this study by the research team was of minimal risk and adverse events were not anticipated. In the unlikely event that such events occurred, the experimental procedure would be terminated and serious adverse events will be reported within 48 h to all relevant stakeholders (including the European Commission and regulatory agencies).

Also, all data collected during the study (both hard copies and electronic) were monitored periodically by EP to ensure maximum safety. Data would be destroyed during the project only if participants or their families specifically require for this to happen. This could be done by contacting EP and requesting data destruction. None of the participating families asked for data destruction to date.

#### Confidentiality and Security of Data

Risks to subject confidentiality were minimized by adopting suitable data storage procedures. Data were/are kept in locked rooms and in locked file cabinets and on password protected computers. More specifically, the consent forms were/are locked in a filing cabinet. Hard copies of the testing protocols and clinical notes were/are also stored in a locked filing cabinet. All hard copies of research data and the clinical information containing personal health information were stored separately in locked file cabinets. Importantly, consent forms were/are NOT be stored with the data so that children are not identifiable by unauthorized sources.

All data for each child were identified by numerical ID; this preserves the anonymity of the child. The master file with children’s names and identification numbers (needed to ensure that children meet eligibility criteria) were/are entered in a password protected excel file on a password protected computer. Only the research team had/has authorized access to the master file. All electronic data are stored on a secure server or password protected computers that are furnished with firewalls and anti-spy and anti-virus software. Names never appeared or will appear in any publication or be mentioned in any public place in connection with this project. The database will be maintained within the existing data management system (i.e., password-protected secure databases) providing a high degree of security and quality monitoring.

#### Data Destruction

Finally, in relation to data destruction the research team complied with the national guidelines for data destruction. In most cases, records are kept for 10 years unless there is a specific request for the data to be destroyed at an earlier point. Therefore, all data are kept for 10 years (unless participants request to be destroyed at another point) and will be destroyed after the collapse of this time frame. Consent givers were informed that it is common in research practice to keep the data for this substantial time to allow full scientific analyses to take place. They were reassured that the data will remain safe, confidential and anonymous during that time and in any form of dissemination. However, they were also informed of their right to request destruction of the acquired data any time during or after the completion of the study.

#### Economic Considerations and Insurance of Participants

Participants received monetary compensation for participation in each session of the experiment. For each behavioral testing/questionnaire session they received 20 Euros ($30). Participants were also be eligible for a 40 Euros ($50) bonus for completion of all study sessions. In detail, children were asked to participate in three visits during which they will receive both the implicit learning tasks and the standardized screening tests and they would be compensated 60 Euros ($90) in total. The study had minimal risks; but in the very unlike event of an injury, children and their families would be fully compensated for any medical costs. There were no such incidents.

#### Benefits of the Study

All participants were provided with the results of standardized behavioral assessment batteries. Other than that, they received no direct benefits to health or well-being and they were made fully aware of that fact before participating. However, our research on implicit learning across different modalities and its relationship with other cognitive abilities such as reading has implications for theories of learning and reading as well as for other didactic and pedagogical aspects of reading.

## Author Contributions

EP conceptualized the study, defined and prepared the experimental stimuli and design, organized, and supervised the recruitment of participants, carried out the data collection, and contributed to the writing of the manuscript. LB carried out all statistical analyses, took care of the interpretation and description of the results, and contributed to the writing of the manuscript. Both authors contributed to the manuscript revision, and read and approved the submitted version.

## Conflict of Interest Statement

The authors declare that the research was conducted in the absence of any commercial or financial relationships that could be construed as a potential conflict of interest.

## Footnotes

1 The term “*implicit statistical learning*” proposed by Conway and Christiansen (2009) is used here to denote the fusion of the two traditions that of implicit learning and of statistical learning; assuming they tap on the same phenomena. However, the study does not adopt any strong stances on the availability to consciousness of the resulting knowledge; the term “implicit” refers to the undirected nature of the learning process.

2 Originally, 33 children were tested. However, two children were excluded from our analysis due to many missing values across all our tasks and measures. Please also note that from our final sample (31), one child did not complete the Auditory ISL task.

3 All children underwent a hearing test.

4 This technique was chosen to maintain attention and ensure that children were engaged with the task. One child who showed clear behavioral signs of frequent disengagement was not included in the analysis.

5 E-Prime Experiment version = 1.0.0.50; Runtime version = 2.0.10.242; Studio version = 147.

6 These BFs, and all BFs reported subsequently, were obtained using JASP Team (2018, Version 0.10), using the default stretched Beta prior with width 1.

7 Note that a large range of measures was included in the test battery (see Table 1), the selection of measures here is motivated by our theoretical considerations as outlined in the section “Introduction.”

8 Since ISL performance is not standardized one could also argue for exploring the correlations with raw scores, reflecting absolute phonological/reading skills. None of the correlations between ISL performance and ISL raw scores reached significance when controlling for multiple comparisons (all *p*’s > 0.11). Looking at these same correlations controlling for age, we observed a pattern of results very similar to the one with standard scores reported in detail below with only the partial correlation between raw phonological awareness score and visual ISL being near-significant (*r* = 0.36, *p* = 0.56; with *p*’s for all other correlations > 0.36).

9 With a low test reliability we can not expect correlations with other outcome measures.
